# High-dose Glycerol Monolaurate Up-Regulated Beneficial Indigenous Microbiota without Inducing Metabolic Dysfunction and Systemic Inflammation: New Insights into Its Antimicrobial Potential

**DOI:** 10.3390/nu11091981

**Published:** 2019-08-22

**Authors:** Qiufen Mo, Aikun Fu, Lingli Deng, Minjie Zhao, Yang Li, Hui Zhang, Fengqin Feng

**Affiliations:** 1 College of Biosystems Engineering and Food Science, Zhejiang Key Laboratory for Agro-Food Processing, National Engineering Laboratory of Intelligent Food Technology and Equipment, Key Laboratory for Agro-Products Postharvest Handling of Ministry of Agriculture and Rural Affairs, Key Laboratory for Agro-Products Nutritional Evaluation of Ministry of Agriculture, Zhejiang University, Hangzhou 310058, China; 2 Ningbo Research Institute, Zhejiang University, Ningbo 315100, China; 3 Institute of Biology, Westlake Institute for Advanced Study, School of Life Sciences, Westlake University, Hangzhou 310064, China

**Keywords:** glycerol monolaurate, gut microbiota, metabolic dysfunction, barrier function, anti-inflammation

## Abstract

Glycerol monolaurate (GML) has potent antimicrobial and anti-inflammatory activities. The present study aimed to assess the dose-dependent antimicrobial-effects of GML on the gut microbiota, glucose and lipid metabolism and inflammatory response in C57BL/6 mice. Mice were fed on diets supplemented with GML at dose of 400, 800 and 1600 mg kg^−1^ for 4 months, respectively. Results showed that supplementation of GML, regardless of the dosages, induced modest body weight gain without affecting epididymal/brown fat pad, lipid profiles and glycemic markers. A high dose of GML (1600 mg kg^−1^) showed positive impacts on the anti-inflammatory TGF-β1 and IL-22. GML modulated the indigenous microbiota in a dose-dependent manner. It was found that 400 and 800 mg kg^−1^ GML improved the richness of *Barnesiella*, whereas a high dosage of GML (1600 mg kg^−1^) significantly increased the relative abundances of *Clostridium XIVa*, *Oscillibacter* and *Parasutterella*. The present work indicated that GML could upregulate the favorable microbial taxa without inducing systemic inflammation and dysfunction of glucose and lipid metabolism.

## 1. Introduction

Glycerol monolaurate (GML) is a nutritional medium-chain fatty acid (MCFA) monoester that is found in human breast milk and coconut oil. According to the Food and Drug Administration, GML was designated as a safe food additive (GRAS); it possesses antibacterial, antiviral and anti-inflammatory functions [1,2,3]. GML was bactericidal for most gram-positive pathogens and some gram-negative bacteria with lipo-oligosaccharides, but completely inactive against lipopolysaccharide-generated Pseudomonas aeruginosa and Enterobacteriaceae [1,3,4]. In addition, GML was safe for chronic use (50 mg mL^−1^) in *Rhesus Macaque* [3] and protected against repeated intravaginal infection by high doses of simian immunodeficiency virus (SIV) [2]. Furthermore, an in-depth clinical study concluded that GML applied on tampons (approximately 8 mg) was of great benefit to vaginal health by reducing *Staphylococcus aureus* exotoxin production and resulting vaginal pro-inflammatory interleukin 8 (IL-8) secretion [5]. What’s more, an investigation into the underlying mechanisms established GML as a potential immunosuppressant for therapeutic applications, including autoimmune, psoriasis and inflammatory bowel disease, based on the effective anti-inflammatory activity and the T cell-suppressed functions of GML [6].

Gut microbiota have a mutualistic relationship with the host [7]. There is a growing appreciation of the role of the gut microbiota and their metabolites in the body’s weight control, metabolic function and immune/inflammatory homeostasis [7,8,9]. Recently, the influence of GML on the gut microbial community has been explored. However, recent data illustrated the paradoxical regulation of gut microbiota by dose and duration of GML feeding. Jiang et al. [10]. reported that the administration of 150 mg kg^−1^ GML for 8 weeks induced microbial dysbiosis and metabolic-related dyslipidemia in healthy mice. In contrast, Li et al. [11] showed that GML (150 mg kg^−1^) intake for 6 weeks did not result in the same microbial change. Furthermore, the nearest finding suggested that a comparatively higher dose of GML supplementation (450 mg kg^−1^) improved metabolic disorder in high fat diet-fed mice by increasing the abundance of beneficial microbiota, such as *Akkermansia*, *Bifidobacterium* and *Lactobacillus* (doi: 10.1002/mnfr.201801417). GML is generally known as a chemically glycerol derivative of a saturated 12 carbon medium chain fatty acid (MCFAs) [12]. Similarly, previous studies have demonstrated that MCFAs caused a dose-dependent microbial change in animals. At low concentrations (about 3.78 mmol kg^−1^), MCFAs acted as modulators of the gastric bacterial ecology without composition shift in weaned piglets [13], while at higher concentrations (i.e., up to 250 mmol), MCFAs exerted mainly antimicrobial activities [13,14,15]. Due to the conflicting outcomes with regards to gut microbiota, the precise functional role of GML on the microbial community needs to be ascertained. Hence, this study aimed to distinguish the modulating effect and the antimicrobial effects of varying doses of GML on indigenous microbiota in mice.

Previous research has indicated that some commonly-used emulsifiers might affect the host (intestinal permeability, colitis and metabolic syndrome) directly or by impacting the intestinal microbiota [16,17]. Given GML is an effective antimicrobial emulsifier, it is important to determine the systemic effects triggered by different doses of GML, including the influence on metabolic functions, intestinal barrier and immune/inflammatory state, as well as on the gut microbiota.

## 2. Materials and Methods

### 2.1. Mice

Male C57BL/6 mice aged 4–5 weeks were purchased from the Shanghai SLAC Laboratory Animal Co. Ltd. (Shanghai, China) and maintained in specific pathogen-free conditions at Laboratory Animal Research Center of Zhejiang Chinese Medical University (Hangzhou, China) under institutionally-approved protocols (ZJU-BEFS-2017002).

### 2.2. Experimental Processes

Mice in the control group (NCD) were fed with a regular chow diet (no. M01-F25), while mice in the experimental groups (G400, G800 and G1600 groups) were fed on customized basal diet incorporated with 400, 800 and 1600 mg kg^−1^ GML (Hangzhou Kangyuan Food Science and Technology Co., Ltd., Hangzhou, China), respectively. The diets were purchased from Shanghai SLAC Laboratory Animal Co. (Shanghai, China). Feed intake for each cage and body weights were determined every week. After 4 months, fresh feces were collected for 16S rRNA gene sequencing and short-chain fatty acids (SCFAs) measurement; blood was also collected and serum was generated by centrifugation (3000× *g* for 20 min at 4 °C). Mice were euthanized and liver weight and adipose fat pads were measured. Organs, including the jejunum, ileum, and epididymal fat were dissected for further analysis.

### 2.3. Histology Analysis

Epididymal fat, duodenum, jejunum, and colon samples were fixed in 10% (*v/v*) buffered formalin at room temperature, embedded in paraffin and then sectioned at 4 μm thickness. Epididymal fat, duodenum and colon sections were stained with haematoxylin and eosin (H&E), and jejunum sections were stained with Alcian-Blue/Periodic acid-Schiff (AB/PAS) to visualize the total mucins at core facilities, Zhejiang University School of Medicine (Hangzhou, China). Images were obtained under a microscope (Leica ICC50W, Wetzlar, Germany). The number and size of the stained epididymal adipocytes were analyzed by the Image J software (National Institutes of Health, Bethesda, MD, USA), and then calculated as the mean size of the 6 images of each sample, and finally, as the average size of each group [11]. The number of mucin-secreted goblet cells was identified by the number of PAS positive cell per villus-crypt axis, as previously described [18].

The morphology of jejunum was characterized by transmission electron microscope (TEM) as previous described [19]. Briefly, a 2-cm-long jejunum specimen was excised and fixed in 2.5% (*w*/*v*) glutaraldehyde in PBS (0.1 M, pH 7.0) overnight at 4 °C and fixed with 1% osmium tetroxide for 2 h. After three rinses with PBS, the specimens were dehydrated, embedded and sectioned in LEICA EM UC7 ultratome (Leica Microsystems, Wetzlar, Germany), and the ultrathin sections were obtained and stained using uranyl acetate and alkaline lead citrate for 5 to 10 min, respectively; samples were observed using a Hitachi Model H-7650 TEM (Hitachi, Tokyo, Japan).

### 2.4. Quantitative Reverse-Transcription PCR (qRT-PCR) Analysis

Ileal samples were determined for the mRNA expression of *muc2*, *zo1*, *occludin*, *claudin-1* and *jam-1*. Total RNAs were isolated using TRIzol (Vazyme Biotech Co., Ltd., Shanghai, China) according to the manufacturer’s instructions. The qRT-PCR analysis was performed using the 2×ChamQ SYBR Color qPCR Master Mix (Vazyme Biotech Co., Ltd., Nanjing, China) in a LightCycler 480 system (Roche, Basel, Switzerland) with specific mouse primers (Supplemental Table S1). The results were normalized to the housekeeping β-actin gene using the 2^–ΔΔCt^ method [20].

### 2.5. Plasma Parameters Analysis

The levels of serum total triglycerides (TG), total cholesterol (TC), low-density lipoprotein-cholesterol (LDL-C), high-density lipoprotein-cholesterol (HDL-C), glutamic-oxaloacetic transaminase (GOT), glutamic-pyruvic transaminase (GOT) and alkaline phosphatase (AKP) were measured using commercial kits (Nanjing Jiancheng Bioengineering Institute, Nanjing, China) according to the respective protocols.

Fasting blood-glucose (fasting Glu), serum insulin, free fatty acid (FFA), adiponectin, leptin, lipopolysaccharide (LPS) and lipopolysaccharide-binding protein (LBP) were measured with the ELISA kit (Cloud-Clone corp., Wuhan, China) according to the prescribed protocols, respectively. HOMA-IR, an index of insulin resistance, was calculated as previous described [21].

Cytokines, including tumor necrosis factor-α (TNF-α), interleukin-6 (IL-6), IL-1β, IL-10, IL-22, IL-12/P70, interferon-γ (IFN-γ) and transforming growth factor β1 (TGF-β1), were analyzed with the ELISA kit (eBioscience, San Diego, CA, USA) according to the manufacturer’s instructions.

### 2.6. Fecal Microbiota Analysis by 16S rRNA Gene Sequencing

Bacterial DNA were extracted from frozen feces using a QIAamp DNA Stool Mini Kit (QIAGEN, Venlo, The Netherlands) according to the manufacturer’s protocol. 16S rRNA paired-end sequencing targeting the V3-V4 hypervariable region was performed using Illumina HiSeq technology at Realbio Technology Inc. (Shanghai, China). 16S rRNA gene sequence analysis was carried out according to previous work [17]. In detail, raw reads in 2 × 250 bp length were merged using the PANDAseq software package (GitHub, Inc. San Francisco, CA, USA) [22], and were assigned to respective sample according to the specific barcodes. The sequences went through quality filtered steps using the Quantitative Insights Into Microbial Ecology software (QIIME, version 1.8.0) [23], including adaptor removal and trimming 3’ bases with quality scores below 20. Reads showing more than three consecutive low-quality base calls were discarded. The processed sequences defined at a 97% similarity threshold were assigned to operational taxonomic units (OTUs), and taxonomical classification was performed using the RDP-classifier with the RDP Release 11.5 database [24].

The OTU absolute abundance table was converted to relative abundances by normalizing to total OTU clustering to analyze the composition and structure of gut microbiota using QIIME, version 1.8.0 [23]. The linear discriminant analysis (LDA) effect size (LEfSe) algorithm was applied to identify specific taxa among different groups.

### 2.7. Short-Chain Fatty Acids Composition Analysis

Short-chain fatty acid composition was analyzed from fecal samples via gas chromatography following the previously-described protocol [25]. Fresh fecal samples were collected from each mouse and weighed; they were then homogenized in 250 μL of ultrapure water for 5 min. The fecal suspension (pH 2–3) was generated by adding 5 M HCl and incubation for 15 min at room temperature with intermittent shaking. After centrifugation at 3000× *g* for 20 min, the resulting supernatant was transferred into a new eppendorf tube, and 2-ethylbutyric acid (TEBA) was supplemented into the supernatant at a final concentration of 1 mmol L^−1^. The Shimadzu GC-2014 system and a column (30 m, 0.53 mm, 0.50 μm) with a free fatty acid phase (DB-FFAP 125–3237, J&W Scientific, Agilent Technologies Inc., Santa Clara, CA, USA) were used for chromatographic analysis.

Nitrogen was the carrier at a flow rate of 15 mL min^−1^. The initial oven temperature was set at 100 °C and maintained for 30 s, increased to 180 °C at a rate of 8 °C min^−1^ and finally, for 60 s, then raised to 200 °C at 20 °C min^−1^ and continued for 15 min. The flame ionization detector and injection port were kept at 240 °C and 200 °C, respectively. The flow rates of hydrogen, nitrogen and air were 30, 20 and 300 mL min^−1^, respectively. The injected volume of each sample for GC analysis was 1 μL, and each analysis had a run period of 27.5 min.

### 2.8. Statistical Analysis

The data were expressed as the mean ± standard deviation (SD), and were analyzed by one-way ANOVA using GraphPad Prism (version 6.0, GraphPad Software Inc., San Diego, CA, USA) followed by Tukey’s multiple-comparison test. A value of *p* < 0.05 indicated a statistically-significant difference.

## 3. Results

### 3.1. Effect of GML on the Body Weight, Feed Intake, Liver Index and Adipocyte size

**Figure 1:**
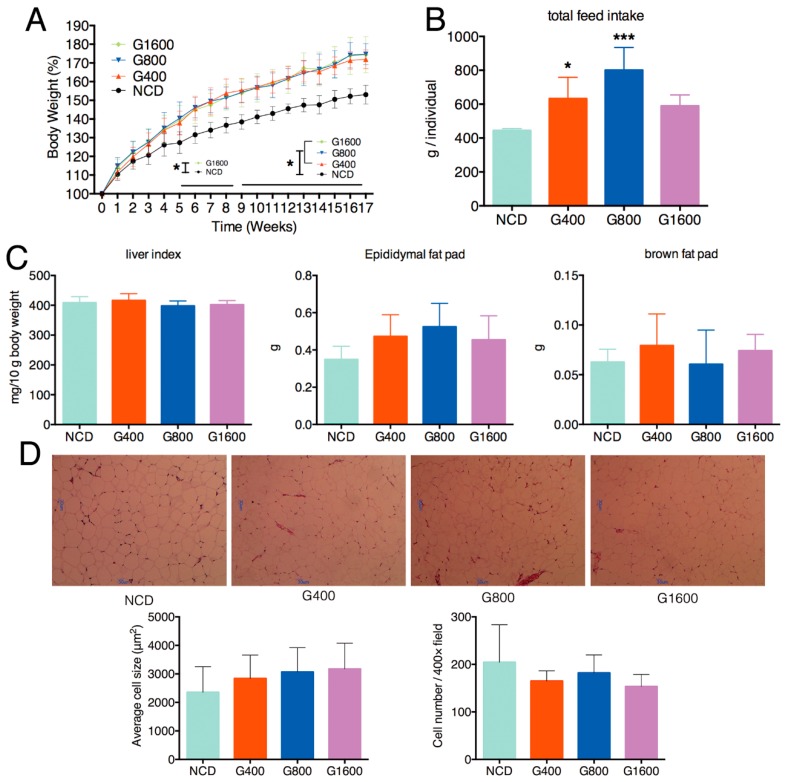
Effect of intake of different doses of GML on the (**A**) body weight, which expressed as a percentage compared to the initial body weight defined as 100%, (**B**) total food intake and (**C**) the liver indices, epididymal fat pad and brown fat pad of mice. (**D**) H&E staining of the epididymal adipose tissue (*n* = 6, magnification: 100×). Data were means ± SD; * *p* < 0.05, *** *p* < 0.001 compared with the NCD group.

Dietary supplementation of GML resulted in significant gains in total weight, regardless of the doses of GML (Figure 1A). The total feed intake in mice fed with 400 and 800 mg kg^−1^ GML showed a marked increase (Figure 1B, *p* < 0.05 and *p* < 0.001, respectively), but there was no significant elevation in the G1600 group (Figure 1B) compared with the NCD group. Despite the body weight gain, GML exposure has no detectable effect on epididymal fat pad, brown fat pad and live index (Figure 1C). Consistent with the result of the unchanged epididymal fat pad, no significant differences on the size of epididymal adipocyte were observed among different groups (Figure 1D).

### 3.2. Effect of GML on the Blood Biochemical Parameters

The effects of GML on the blood biochemical parameters are shown in Table 1. A diet containing 400 mg kg^−1^ GML led to a significant increase in TG. In contrast, adding 1600 mg kg^−1^ GML into diets tended to downregulate the plasma LDL-C level and upregulate the plasma HDL-C concentration. Consequently, a trend of reduction was observed in the G1600 group in terms of the LDL-C/HDL-C ratio when compared with the NCD group. GML exerted no significant effect on plasma TC and atherogenic index (TC − HDL-C)/HDL-C). Mice exposed to different doses of GML showed no obvious changes in the amounts of fasting Glu, insulin, adiponectin, leptin, FFA, GOT, GPT and AKP, relative to the NCD group.

### 3.3. Effect of GML on the Histological Feature and Barrier Function of Intestine

**Figure 2:**
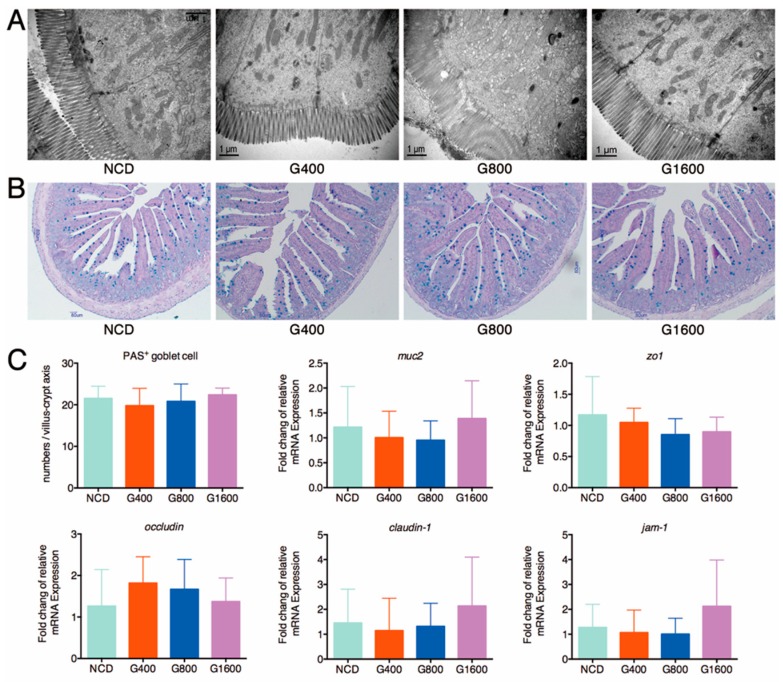
Effect of different doses of GML on the intestinal barrier function. (**A**) TEM analysis of the jejunum section, (**B**) AB/PAS staining of jejunum to define the secretion of mucus of goblet cells, (**C**) The mRNA levels of different genes (*muc2*, *zo1*, *occludin*, *claudin-1* and *jam-1*) in the distal ileum measured using qRT-PCR and normalized to the β-actin mRNA expression. Data were expressed as mean ± SD (*n* = 6–8).

A detailed histological examination of the duodenal and colonic tissue revealed that the global features were unchanged, including the intact epithelial layer and the cell membrane, normal mucosal thickness and well-organized villi and crypt (Supplemental Figure S1). An ultrastructural analysis of the jejunum specimens showed that the rows of epithelial cells were organized closely with narrow paracellular spaces; the microvilli on the cell surface were well-arranged, and the marginal zone of tight junction was clear and complete in the GML-treated mice, similar to the morphological characteristics of the jejunum in the NCD group (Figure 2A). It is important to highlight that GML treatment did not affect the total mucins secretion, as the numbers of PAS-positive goblet cells in the jejunum of GML-treated mice were comparable to the NCD group (Figure 2B,C). Goblet cells generate mucins, mainly Muc2, which constitute the first line of the intestinal barrier [26]. Similarly, there was no significant difference in the mRNA expression of *muc2*, *zo1*, *occludin*, *claudin-1* and *jam-1* in the ileal section among the different groups (Figure 2C), indicating that GML maintained the integrity of histological features and barrier function of intestine.

### 3.4. Effect of GML on Serum Inflammatory-Related Parameters

The circulating levels of the pro-inflammatory factors TNF-α, IL-6, IL-1β, IFN-γ, IL-12/p70, LPS and LBP showed no obvious difference between the NCD- and GML-treated groups (Table 2). The administration of 1600 mg kg^−1^ GML, rather than 400 mg kg^−1^ and 800 mg kg^−1^, increased the circulating levels of anti-inflammatory cytokines including TGF-β1 and IL-22, but not for IL-10 (Table 2). The results revealed that a high dose of GML may contribute to the promotion of an anti-inflammatory environment instead of inducing systemic inflammation.

### 3.5. Effects of GML on Fecal Microbiota

**Figure 3:**
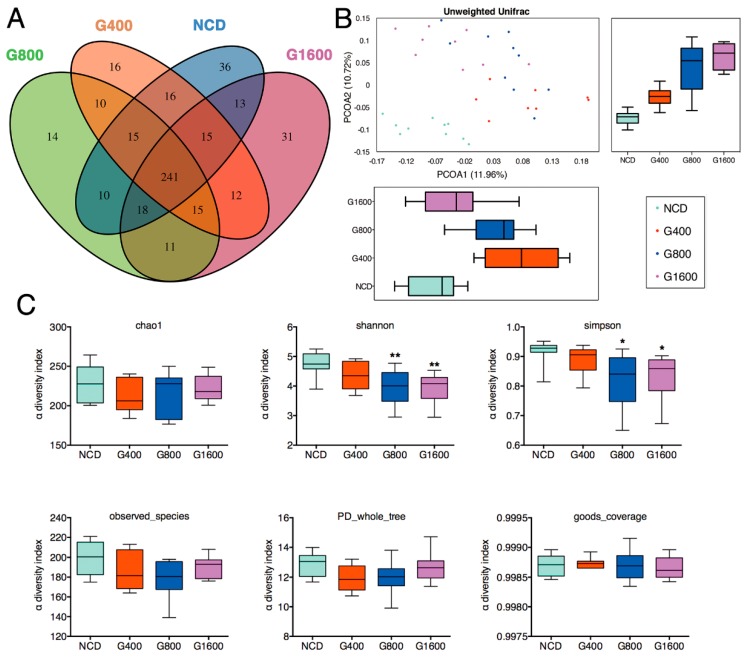
Change in fecal microbiota in mice fed with different doses of GML-supplemented diets. (**A**) Venn diagram of OTUs. (**B**) β-diversity analysis was performed by using the unweighted version of the UniFrac-based PCoA. (**C**) α-diversity analysis based on indices of chao1, Shannon, Simpson, Observed species, PD whole tree and goods coverage. Data were means ± SD (*n* = 8–10); * *p* < 0.05 and ** *p* < 0.01 compared with the NCD group.

Venn diagrams demonstrated that 241 OTUs existed among all four groups, and that 36, 16, 14 and 31 specific OTUs were unique to the NCD, G400, G800 and G1600 group, respectively (Figure 3A). A beta diversity analysis revealed a distinct clustering of fecal microbiota among the NCD and GML-treated groups, as calculated by unweighted UniFrac principal coordinates analysis (PCoA) (Figure 3B). Specifically, supplementation of GML shifted the microbial composition away from that of the NCD group in PC2, which explained the observed 10.72% of total variance. PC1 showed that the microbiota in the G400 and G800 groups separated from the NCD group, whereas the microbiota between the NCD and G1600 groups were not divided (Figure 3B). No significant differences were observed for indices of Chao1, goods coverage, observed species, and PD whole tree among the four groups. However, the Simpson and Shannon indices in higher-dose GML groups (800 and 1600 mg kg^−1^) were lower than those in the NCD group (Figure 3C).

**Figure 4:**
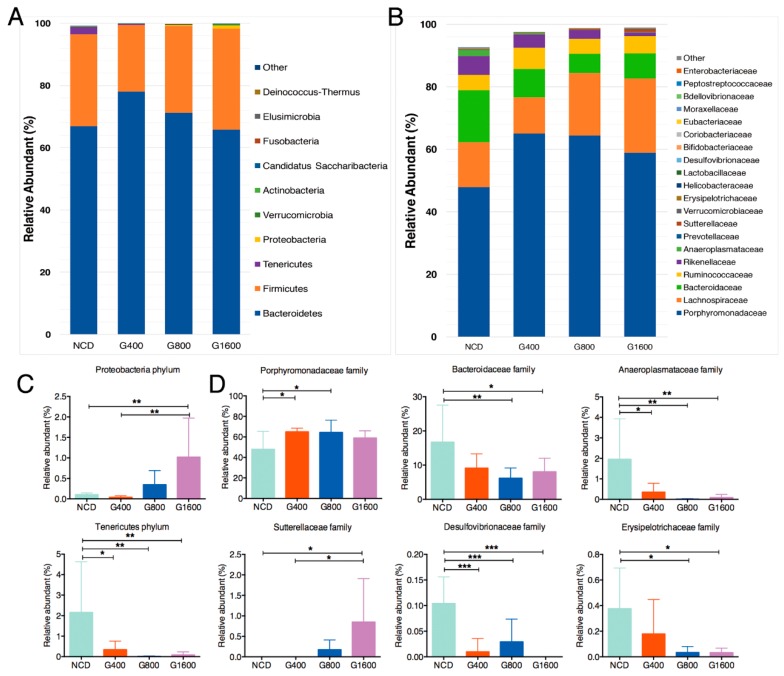
GML altered the fecal microbiota composition in mice. Microbial taxonomic profiling in the (**A**) phylum level and (**B**) family level among different groups, and different colors represent different taxa. Relative abundance of the bacterial (**C**) phylum and (**D**) families that differentially detected in fecal samples. Results were expressed as mean ± SD (*n* = 8–10), * *p* < 0.05; ** *p* < 0.01; *** *p* < 0.001.

Taxonomic profiling indicated that supplementation of GML led to a decrease in the phylum *Tenericutes* (Figure 4A,C), especially in the family *Anaeroplasmataceae* (Figure 4B,D). However, 1600 mg kg^−1^ GML induced a sharp increase in the *Proteobacteria* content, compared to the NCD and G400 groups (Figure 4A,C). Within the phylum *Proteobacteria*, significantly increased *Sutterellaceae* in the G1600 group and decreased *Desulfovibrionaceae* in all GML-treated groups were observed at the family level (Figure 4B,D). Adding GML to the diet had no effect on the relative richness of phylum *Firmicutes* and *Bacteriodetes* (Figure 4A). Regarding the family levels, *Porphyromonadaceae* was significantly elevated in the G400 and G800 groups, while *Bacteroidaceae* and *Erysipelotrichaceae* were markedly declined in the G800 and G1600 groups relative to the NCD group (Figure 4B,D).

**Figure 5:**
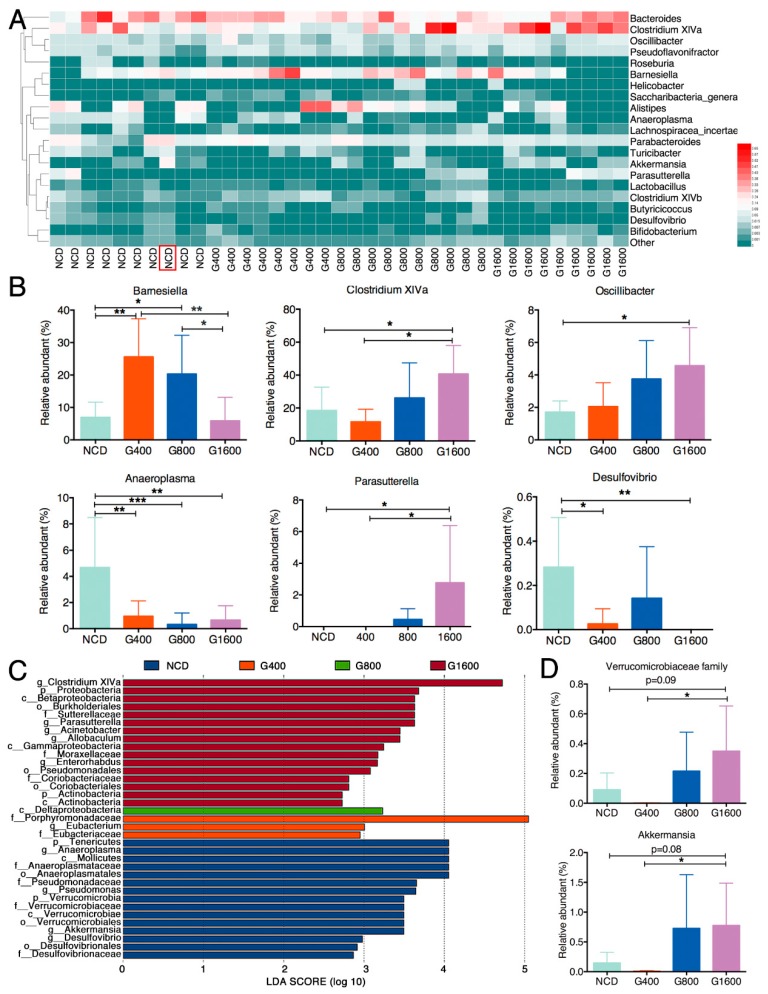
GML altered the fecal microbiota composition in mice. (**A**) The heat map of the relative abundance of the top 20 genera among different groups. The red color indicates high values while the green color means low values. (**B**) Relative abundance of the bacterial genera that differentially detected in fecal samples among different groups. (**C**) a total of 35 taxa showed significant differences in their relative abundance among the NCD, G400, G800 and G1600 groups with 2.5 as LDA score threshold. (**D**) The relative abundance of genus *Akkermansia* among four groups after eliminating the outlier which were detected with the ROUT method (Q = 1%) using GraphPad Prism Version 6. Results were expressed as mean ± SD (*n* = 8–10), * *p* < 0.05; ** *p* < 0.01; *** *p* < 0.001.

To illustrate the specific changes in the microbial taxa, we further analyzed the relative abundance of the 20 predominant genera in the four groups. The results showed that the 400 and 800 mg kg^−1^ GML-treated groups showed a significantly higher level of *Barnesiella*. However, the 1600 mg kg^−1^ GML-treated group had a similar abundance of *Barnesiella* as the NCD group (Figure 5A,B). Of note, GML dose-dependently boosted the relative abundant of *Clostridium XIVa* and *Oscillibacter*, which reached statistical significance at high dosage (1600 mg kg^−1^) relative to the NCD group (Figure 5A,B). Within the phylum *Proteobacteria*, *Parasutterella* notably thrived (*p* < 0.05), but *Desulfovibrio* was not detected in the G1600 group compared with the NCD group. As expected, we found that the level of *Anaeroplasma* in all the GML-treated groups was considerably lower than the NCD group (Figure 5A,B). The collective results demonstrate that although several particular deviations were observed, GML showed a dose-dependent capacity to modulate the microbial communities, as 400 and 800 mg kg^−1^ GML regulated some taxa at the family and genus levels, while 1600 mg kg^−1^ GML operated a more in-depth reconstruction of the microbial community.

### 3.6. Effect of GML on Phylotypes

Linear discriminant analysis effect size (LEfSe) analysis was conducted to identify the key bacterial phylotypes that were differentially represented among the four groups. As showed in Figure 5C, mice in the NCD group showed a higher abundance of phylum *Tenericutes*, as well as the homologous lower branches *Mollicutes*, *Anaeroplasmatales*, *Anaeroplasmataceae*, *Anaeroplasma*. Furthermore, the Pseudomonadaceae (*Pseudomonas*) and *Desulfovibrionaceae* (*Desulfovibrio*) predominated in the NCD group. The G400 group showed enrichment of family *Porphyromonadaceae* and *Eubacteriaceae* (mainly genus *Eubacterium*). Only *Deltaproteobacteria* was observed to be significantly abundant in the G800 group. Of note, *Clostridium XIVa* was significantly overrepresented in the G1600 group. It was found that 1600 mg kg^−1^ GML led to the enrichment of phylum *Proteobacteria*, class *Betaproteobacteria* and *Gammaproteobacteria*, order *Burkholderiales* and *Pseudomonadales*, family *Sutterellaceae* and *Moraxellaceae*, as well as genera *Parasutterella* and *Acinetobacter* (Figure 5C). However, we observed one exception as well: the LEfSe analysis showed that the genus *Akkermansia* within the family *Verrucomicrobiaceae* was identified as being enriched in the NCD group. However, this effect was caused by a single outlier, shown in Figure 5A (marked with red box). Hence, we measured the relative abundance of genus *Akkermansia* among the four groups (Figure 5D) after eliminating the outlier which was detected with the ROUT method (Q = 1%). The result showed that the richness of *Akkermansia* declined in the G400 group compared with the NCD group, although no statistical difference was observed (0.146% in the NCD group vs. 0.007% in the G400 group). However, the abundance of *Akkermansia* increased with increasing the dose of GML.

### 3.7. Effects of GML on Fecal Short Chain Fatty Acid

The effect of GML on the concentrations of fecal short-chain fatty acid (SCFAs) is summarized in Table 3. As expected, the most abundant SCFAs in feces were acetic acid, followed by propionic acid and butyric acid. Adding GML into the diet decreased the production of fecal acetic acid by a wide margin, but had no evident effect on other SCFAs, including propionic acid, butyric acid, isobutyric acid, isovaleric acid valeric acid and hexanoic acid. Consequently, total SCFAs showed a significant reduction in the G400 and G800 groups, and tended to decrease in the G1600 group.

## 4. Discussion

In the present study, we fed mice with different dosages of GML following years of experiments with GML as an antimicrobial-emulsifier under different circumstances [3,6,10], and aimed to provide more practical and relevant information on the modulating effect and the antimicrobial effects of GML with regard to gut microbiota.

According to the Shannon and Simpson indices, supplementation with a relatively high dose of GML led to a decrease in microbial diversity. GML was known to have a wide antibacterial spectrum, especially at higher concentrations [3]. The decrease in the α-diversity in the G800 and G1600 groups may be related to bactericidal effect of GML. The results support the hypothesis that a low dose of GML may act as modulator of the gut microbiota, while a higher dose introduces its antimicrobial potential.

GML-supplemented diets affected the gut microbial structure, with reduced abundance of phylum *Tenericutes* (genus *Anaeroplasma*) and dose-dependently elevated levels of phylum *Proteobacteria*. The result was different from the previous finding that emulsifier carboxymethylcellulose leaded to an increase in *Anaeroplasma* [27]. Mice in the G400 and G800 groups, but not the G1600 group, harbored significantly greater abundances of *Barnesiella*, which was positively associated with a healthy state [28], and may confer anti-inflammatory properties in the context of DSS challenge [29]. In the G1600 group, the augmentation of the phylum *Proteobacteria* was mainly due to the genus *Parasutterella* within the family *Sutterellaceae* and the genus *Acinetobacter* within family *Moraxellaceae*, as determined by LEfSe analysis. *Parasutterella* has been defined as a taxon of the healthy core microbiota in the human gut [30]. Several trials have highlighted that *Parasutterella* showed a negative correlation with high fat diet-induced metabolic disorder in mice [31,32] and with hypothalamic inflammation in humans [33]. Consistent with the favorable effect of GML on *Parasutterella* content, supplementations of prebiotic or resistance starch, which were proposed as approaches to polarize the gut microbiota to a beneficial community [34], have the same positive effect on the richness of *Parasutterella*. In addition, *Acinetobacter* has been recognized as a specific core species that resides in the colonic crypts of healthy animals [35]. Despite the high content of *Proteobacteria* in the G1600 group, GML supplement markedly reduced the quantity of family *Desulfovibrionaceae* and genus *Desulfovibrio*, which have been linked to adverse effects on host health, such as producing toxic sulphide, which contributes to the development of ulcerative colitis [36], and inducing LPS endotoxemia [37]. Mice in the G1600 group exhibited higher levels of *Clostridium XIVa* and *Oscillibacter*, which have been shown to have anti-inflammatory functions and play crucial roles in the maintenance of mucosal homeostasis [38]. In the preceding evidence, most gram-positive pathogens were susceptible to the bactericidal effects of GML [3]. The antigram-positive germs effect of GML may open up ecological niches for occupation by *Clostridium XIVa* and *Oscillibacter* in a dose-dependent manner. In contrast to *Desulfovibrio*, both *Oscillibacter* and *Barnesiella* showed negative associations with LPS [39]. A previous report suggested that 150 mg kg^−1^ GML could significantly decrease *Akkermansia* muciniphila [10]. In the present study, 400 mg kg^−1^ GML caused a similar decline in the level of *Akkermansia*; however, GML rescued and promoted the richness of *Akkermansia* with increasing the dose of GML. Therefore, the outcomes in the current study deepen our understanding of the dose-dependent regulation of GML on the compositions of gut microbiota.

Accumulating reports showcase that adding other commonly-used emulsifiers to diets tends to be linked to the erosion of the protective mucus, the increase of gut permeability, the induction of low-grade inflammation and metabolic disorders in mice [17,34]. Hence, in the present study, the properties of the different amounts of GML-supplemented diets on the host health were investigated by measuring various parameters related to glucose and lipid metabolism, barrier function and inflammation.

Hyperlipidemia is a metabolic disorder with abnormally elevated contents of TC, TG and LDL-C, and with a reduced concentration of HDL-C in the blood [40]. In our work, 1600 mg kg^−1^ GML reduced the concentration of LDL-C by half, compared to the NCD group, and had 36% higher level of HDL-C than the NCD group, though no statistical difference was observed. Furthermore, the LDL-C/HDL-C ratio, which is regarded as an accurate predictor of arteriosclerosis and stroke risk [41], were decreased in the G1600 group at a trend level. The outcomes disagreed with previous studies which reported that the administration of GML at a dosage of 150 mg kg^−1^ for no more than 8 weeks caused a significant changed in serum lipids, especially HDL-C and LDL-C [10,11]. In contrast, the present findings paralleled the results of previous two studies, in which long-term intervention with a coconut oil-rich diet resulted in lower fasting LDL-C concentrations [42,43]. GML is known as a naturally-occurring monoglyceride of coconut oil [44]. The inconsistent results between our study and previous studies could be largely due to the variations in feeding dosages and duration. Another possible explanation was that, in the G1600 group, the increase in the relative abundance of *Parasutterella* and *Oscillibacter* may be associated with the lipid profiles. Two previous studies revealed that *Parasutterella* showed positive correlation with the serum HDL-C levels [45,46]. Li et al. indicated that *Oscillibacter* showed an opposite correlation to serum LDL-C [47].

Hyperglycemia is accompanied by a high fasting glucose level, and is triggered by long-term insulin resistance. Both insulin resistance and hyperglycemia are the main initiators of metabolic features [48]. In the present trial, no significant changes were observed in the levels of glycemic markers, such as fasting blood glucose and insulin, as well as HOMA-IR score. The results were in line with a previous report which demonstrated that no obvious changes in the level of the serum fasting blood glucose, insulin and HOMA-IR were noted between 150 mg kg^−1^ GML-treated group and the control group [10]. In addition, Perry et al. reported that the elevated plasma and fecal levels of microbiota-derived acetate can disrupt glucose/insulin homeostasis, and thus, contribute to metabolic disorder by mediating the gut-brain axis [49]. In our study, the significant reduction in the case of fecal acetate may be a desired health benefit which maintains the glucose/insulin balance.

The levels of leptin and adiponectin, two adipocyte-secreted hormones [50], in the GML-supplemented groups were similar to those of the NCD group. No increase in the serum FFA, which was proved to be released from larger adipocytes [21], was observed in the GML-treated groups. The unchanged concentrations of leptin, adiponectin and FFA were in agreement with the unaffected epididymal fat pad, as well as the epididymal adipocyte size in the present study. Thus, we conclude that the diets supplemented with different amounts of GML did not lead to hyperglycemia and insulin resistance.

What’s more, intact morphology of the jejunum, the unchanged number of mucus-secreted goblet cells and the normal expressions of the barrier-related genes, viz., *zo1*, *occludin*, *claudin-1*, *jam-1* and *muc2* in the ileum after feeding of GML-added diets were highlighted in the current study, suggesting the possible role of GML in the maintenance of mucosal barrier and intestinal health. Similarly, the administration of carrageenan (0.02%) and monoglyceride and diglyceride containing 16 and 18 carbons (0.1%) in dairy cream was reported to promote the expression of *zo1*, *occludin* and *muc2* [18]. Furthermore, in this study, we found no clear alteration in the levels of serum inflammatory markers, such as IL-6, TNF-α, IL-1β, IL-12/p70, IFNγ, LPS and LBP. The same was true for serum anti-inflammatory cytokine IL-10. Instead, the concentrations of serum anti-inflammatory factors, including TGF-β1 and IL-22, were statistically increased in the G1600 group. Extensive evidence revealed that endogenous IL-22 also played an important role in counteracting uncontrolled or chronic inflammation and maintaining homeostasis [51,52]. The pleiotropic functions of IL-22 have been reviewed elsewhere [53]. TGFβ1 is an anti-inflammatory cytokine that regulates many biological and physiological processes including cell proliferation and differentiation, immune response and inflammation [54]. Hence, the positive effects of GML at dose of 1600 mg kg^−1^ on the increased production of anti-inflammatory cytokines can be considered as a favorable benefit.

## 5. Conclusions

The present study provided further insights into the antimicrobial effect of GML from the aspect of the indigenous microbiota. A low dose of GML (400 mg kg^−1^) can be regarded as modulator of gut microbiota without obviously affecting the microbial diversity, whereas a high dose, especially 1600 mg kg^−1^, exerted antimicrobial activity, and consequently allowed specific profitable taxa to flourish. Of importance, most of the metabolic-related parameters, including blood lipid profiles, glucose metabolism and systemic inflammation, as well as intestinal barrier function, were not adversely regulated by GML. Collectively, the current work provided a comprehensive appraisal of the effects of GML on the gut microbiota and host metabolic function; the functional role of GML on gut microbiota as a modulator or antimicrobial compound inspired the further application of GML in microbial dysbiosis-related disorders for preventive purposes.

## Figures and Tables

**Table 1:** Effect of GML on the metabolic-related biochemical parameters.

Parameters	NCD	G400	G800	G1600
TC (mmol L^−1^)	3.71 ± 0.47	3.81 ± 0.76	3.91 ± 0.86	3.66 ± 0.53
TG (mmol L^−1^)	0.82 ± 0.08	1.21 ± 0.25 *	1.03 ± 0.19	0.85 ± 0.32
LDL-C (mmol L^−1^)	0.93 ± 0.56	0.88 ± 0.37	0.75 ± 0.51	0.42 ± 0.34
HDL-C (mmol L^−1^)	1.75 ± 0.39	1.99 ± 0.38	1.88 ± 0.51	2.38 ± 0.77
LDL-C/HDL-C	0.69 ± 0.34	0.54 ± 0.13	0.56 ± 0.25	0.29 ± 0.06 #
atherogenic index	1.20 ± 0.45	0.92 ± 0.20	1.28 ± 1.01	0.60 ± 0.30
fasting Glu (mmol L^−1^)	3.43 ± 0.72	4.54 ± 0.51	4.77 ± 1.47	4.39 ± 0.65
insulin (mU L^−1^)	7.20 ± 1.26	6.36 ± 0.69	6.93 ± 0.85	5.85 ± 0.53
HOMA-IR score	1.09 ± 0.27	1.28 ± 0.11	1.49 ± 0.56	1.14 ± 0.18
adiponectin (pg mL^−1^)	16.32 ± 3.23	13.03 ± 4.51	18.06 ± 2.93	14.13 ± 3.31
leptin (pg mL^−1^)	70.20 ± 16.82	63.51 ± 7.17	99.44 ± 43.24	97.51 ± 43.70
FFA (mmol L^−1^)	145.85 ± 70.79	89.77 ± 25.73	102.05 ± 20.60	125.48 ± 28.36
GOT (U L^−1^)	12.70 ± 2.46	14.73 ± 4.86	16.64 ± 4.76	14.40 ± 2.72
GPT (U L^−1^)	11.83 ± 10.26	15.15 ± 5.94	17.04 ± 2.00	15.52 ± 2.60
AKP (U L^−1^)	7.95 ± 0.85	6.77 ± 1.23	7.27 ± 1.39	8.76 ± 1.51

Data were expressed as mean ± SD (*n* = 6–8). # 0.05 < *p* < 0.1, * *p* < 0.05 vs. NCD group.

**Table 2:** Effect of GML on the serum anti-inflammatory and pro-inflammatory factors.

Parameters	NCD	G400	G800	G1600
TGF-β1 (ng mL^−1^)	14.34 ± 6.98	14.08 ± 7.11	13.72 ± 7.25	23.32 ± 8.87 *
IL-22 (pg mL^−1^)	25.42 ± 5.03	23.63 ± 1.60	21.52 ± 1.31	31.09 ± 2.59 *
IL-10 (pg mL^−1^)	269.21 ± 131.29	369.26 ± 149.80	219.96 ± 183.82	147.65 ± 43.92
TNFα (pg mL^−1^)	22.23 ± 6.24	17.99 ± 7.98	19.63 ± 5.87	20.66 ± 7.63
IL-6 (pg mL^−1^)	28.95 ± 8.05	24.56 ± 8.01	29.19 ± 13.95	26.90 ± 12.56
IL-1β (pg mL^−1^)	13.20 ± 10.23	4.57 ± 3.80	8.03 ± 6.94	24.21 ± 12.26
IL-12/p70 (pg mL^−1^)	33.99 ± 9.78	32.10 ± 10.70	39.12 ± 14.85	37.95 ± 6.43
IFNγ (pg mL^−1^)	36.70 ± 2.00	38.97 ± 2.91	39.86 ± 3.33	40.28 ± 2.62
LPS (U mL^−1^)	11.18 ± 0.44	9.92 ± 4.72	11.11 ± 0.93	10.53 ± 0.77
LBP (μg mL^−1^)	1.59 ± 1.19	1.54 ± 0.81	1.55 ± 0.47	1.53 ± 0.58

Values were expressed as mean ± SD (*n* = 6–8). * *p* < 0.05 vs. NCD group.

**Table 3:** Effect of GML on the concentrations of fecal SCFAs.

Parameters	NCD	G400	G800	G1600
acetic acid	98.15 ± 5.03	68.73 ± 12.14 **	64.95 ± 5.17 ***	75.84 ± 12.55 **
propionic acid	6.97 ± 2.15	6.83 ± 0.64	7.79 ± 1.39	6.60 ± 0.99
butyric acid	5.93 ± 5.42	3.33 ± 0.93	6.72 ± 3.65	7.90 ± 4.11
isobutyric acid	0.60 ± 0.30	0.68 ± 0.27	0.79 ± 0.30	0.82 ± 0.27
valeric acid	0.84 ± 0.35	0.63 ± 0.29	0.83 ± 0.42	0.68 ± 0.33
isovaleric acid	1.19 ± 0.30	0.96 ± 0.17	1.29 ± 0.33	1.37 ± 0.31
hexanoic acid	0.30 ± 0.09	0.35 ± 0.04	0.36 ± 0.07	0.43 ± 0.18
total SCFAs	113.98 ± 12.83	81.50 ± 11.75 **	82.73 ± 8.46 **	93.65 ± 15.05

Data were expressed as mean ± SD (*n* = 6–8). ** *p* < 0.01, *** *p* < 0.001 vs. NCD group.

## Supplementary Materials

The following are available online at https://www.mdpi.com/2072-6643/11/9/1981/s1, Figure S1: H&E staining of the (A) duodenum and (B) colon section from mice treated with different doses of GML to study inflammation of the intestine (*n* = 6), Table S1: Primer sequences used in qRT-PCR assays.
