# Supplementary material for: High-dose Glycerol Monolaurate Up-Regulated Beneficial Indigenous Microbiota without Inducing Metabolic Dysfunction and Systemic Inflammation: New Insights into Its Antimicrobial Potential
Source: Nutrients. 2019 Aug 22;11(9):1981. doi: 10.3390/nu11091981 (PMC6770898; doi:10.3390/nu11091981)
Supplement: Supplementary file 1 [file nutrients-11-01981-s001.pdf]

Table S1. Primer sequences used in qRT-PCR assays.

Genes	Sequence (5′-3′)
muc2-F	ACCGCCTCACTCTGCCCCAA
muc2-R	TGCAGCCACTGCCCGTGATG
zo1-F	AGGACACCAAAGCATGTGAG
zo1-R	GGCATTCCTGCTGGTTACA
occludin-F	AGGAGTTAACGTCGTGGACCGG
occludin-R	GGCAGGAATGCTGTCATTTGCAG
claudin-1-F	GTTTGCAGAGACCCCATCAC
claudin-1-R	AGAAGCCAGGATGAAACCCA
jam-1-F	CCCCGAGTGGAGTGGAAGTTCG
jam-1-R	GAGGTCTGTTTGAATTCCCCCTC

Abbreviations are as follows: *muc2*, mucin 2; *zo1*, zonula occludens-1; *jam-1*, Junctional adhesion molecule 1.

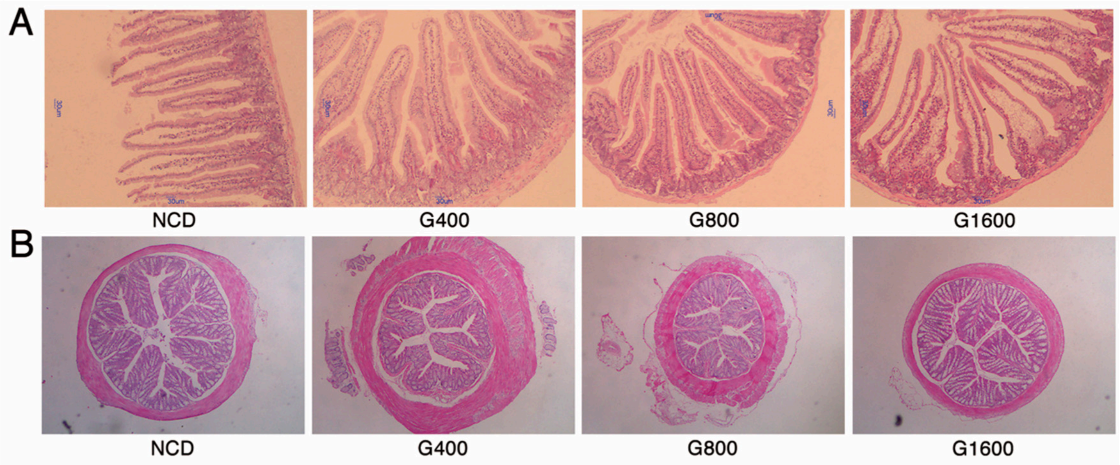

Figure S1. H&E staining of the (A) duodenum and (B) colon section from mice treated with different doses of GML to study inflammation of the intestine ($n = 6$).
